# A Clinical Monitoring Program of COVID-19 Outpatients: A Prospective Cohort Study

**DOI:** 10.1155/2021/6644570

**Published:** 2021-07-20

**Authors:** Hossein Kasiri, Cyrus Mahjub, Mohammadreza Mazaeri, Fahimeh Naderi-Behdani, Aliyeh Bazi, Monireh Ghazaeian, Sahar Fallah

**Affiliations:** ^**1**^ Department of Clinical Pharmacy, Faculty of Pharmacy, Mazandaran University of Medical Sciences, Sari, Mazandaran, Iran; ^2^Department of Emergency, Ibne Sina Hospital, Mazandaran University of Medical Sciences, Sari, Iran; ^3^Department of Clinical Pharmacy, Faculty of Pharmacy, Zabol University of Medical Sciences, Zabol, Iran; ^4^Pharmaceutical Research Center, Department of Clinical Pharmacy, Faculty of Pharmacy, Mazandaran University of Medical Sciences, Sari, Iran; ^5^Department of Biostatistic, Ibne Sina Medical and Educational Center, Mazandaran University of Medical Sciences, Sari, Mazandaran, Iran

## Abstract

**Purpose:**

Coronavirus disease 2019 (COVID-19) has been associated with a high rate of mortality and morbidity. While a high portion of COVID-19 patients have mild symptoms, a limited number of clinical trials have evaluated the clinical course of this large group of patients. This study was designed to investigate the demographics and clinical characteristics and comorbidity of nonhospitalized COVID-19 patients.

**Methods:**

This prospective, observational cohort study was performed on nonhospitalized adult patients (≥18 years) with COVID-19. Pharmacotherapy service was responsible for patients' assessment for up to 1 month. Demographic characteristics, the onset of symptoms, severity, duration, laboratory data, and hospitalization rate were evaluated by a pharmacist-based monitoring program.

**Results:**

From 323 patients who had been referred to the emergency department, 105 individuals were recruited between April 26 and August 2, 2020. Most of the patients were female (66.7%) with a mean age of 39.39 years (SD: ± 15.82). The mean time of the symptom onset was 5.6 days (SD: ±1.79). The majority of patients suffered from fatigue (78.1%), sore throat (67.6%), cough (60%), and myalgia (55.2%). C-reactive protein, white blood cell, lymphocyte, neutrophil-to-lymphocyte ratio, platelet-to-lymphocyte ratio, and hemoglobin levels were recovered significantly during the first two weeks (*P* < 0.001). Hydroxychloroquine, naproxen, diphenhydramine, azithromycin, and vitamin D3 were the most common medications administered (98%, 96%, 94%, 68%, and 57%, respectively). Forty patients were not symptom-free after the one-month follow-up, and 8 patients (7.6%) were required to revisit without the need for hospitalization. Anosmia (18.1%) and fatigue (17.1%) were the most common persisted symptoms. There were no significant differences between symptom-free and symptomatic patients.

**Conclusion:**

Mild COVID-19 patients had a wide variety of symptoms and could be symptomatic even one month after the onset of symptoms. The pharmacist-based monitoring system can contribute beneficially to patients through the evaluation of symptoms, reduction of unnecessary visits, and provision of updated information to patients concerning the status of their illness.

## 1. Introduction

On March 11, 2020, the World Health Organization (WHO) officially announced the outbreak of a new strain of the coronavirus, causing the “novel” coronavirus disease 2019 (abbreviated “COVID-19”) [1]. The first cases of COVID-19 were observed in Wuhan, China, and quickly spread beyond China's borders, affecting 216 countries [2, 3]. The most recent situation report of the Worldometer database states that COVID-19 has already infected as many as 113,501,273 people from 221 countries up to February 25, 2021, causing 2,517,549 deaths [3]. This is the third outbreak of the coronavirus family after the severe acute respiratory syndrome (SARS) and the Middle East respiratory syndrome (MERS) in the past 20 years [4]. After the WHO announced the COVID-19 pandemic a public health emergency of international concern, extensive efforts have been made to control and prevent the disease worldwide [5]. Several risk factors such as advanced age, underlying disease, smoking, obesity, and male sex have been reported among hospitalized patients with COVID-19 and related to disease severity [6–8]. Despite limited data on COVID-19 outpatients, it seems their clinical characteristics and risk factors differ from those of the hospitalized people with the same condition [9, 10]. Moreover, hundreds of clinical trials aiming to find a definitive treatment for COVID-19 have been unsuccessful to date [11, 12].

The high rate of spread and mortality of the severe acute respiratory syndrome coronavirus 2 -(SARS-CoV-2)induced COVID-19 pandemic has exerted great pressure on clinicians and pharmacists throughout the world. While most of the infected patients are not hospitalized [9, 10], characteristics of their clinical course and probable risk factors of symptom deterioration need to be evaluated by the healthcare system during self-isolation.

This cohort study utilized a pharmacotherapy service to monitor and assess nonhospitalized COVID-19 patients in terms of clinical symptoms, disease course, and the need to refer to medical centers during the self-isolation period.

## 2. Methods

### 2.1. Study Setting

This was a prospective, observational cohort study conducted between April 26 and August 2, 2020, in Ibne Sina Hospital, affiliated to the Mazandaran University of Medical Sciences (MAZUMS) in Sari, North of Iran. The study was approved by the MAZUMS Institutional Review Board and Committee for Research Ethics (IR.MAZUMS.REC.1399.109). Written informed consent forms were signed by all the patients.

### 2.2. Participants' Characteristics

Adult patients aged 18 years or more were included in our study if they had mild symptoms of COVID-19, with positive findings compatible with COVID-19 in the lung CT scan or a positive PCR test for SARS-CoV-2. Patients were excluded if they had a moderate-to-severe COVID-19 disease (dyspnea, O2 saturation < 94%, and respiratory rate ≥ 30) [6], and a history of previous probable COVID-19 infection. Moreover, they were excluded if they refused to participate in the study.

### 2.3. Procedure and Data Acquisition

Demographic characteristics of patients including age, sex, underlying diseases, medications used, laboratory tests, and clinical symptoms of the disease were recorded at baseline. A trained pharmacist delivered an educational form containing the WHO recommendations on the principles of home quarantine for patients [13] to the patient as well as a patient self-assessment questionnaire during the period of the study.

The pharmacotherapy service, including a clinical pharmacist and a trained pharmacist, evaluated patients daily by phone calls for the first 14 days after recruitment and then weekly until one month. The monitoring program involved a self-assessment questionnaire that assessed any severe symptoms (such as shortness of breath, confusion, signs of hypoxia, or persistent chest pain or pressure), the need for contacting other clinicians (e.g., a primary care physician or specialist), the clinical course of the disease (recovery or nonrecovered symptoms), and the occurrence of any possible side effects of therapeutic regimens.

The time of symptom onset, symptoms' severity according to VAS (visual analogue scale), course and duration, laboratory data, and the need for hospitalization were recorded during the follow-up period.

### 2.4. Statistics

Statistical analysis was performed using SPSS 24.0 (IBM Corp., Armonk, NY). Frequencies were calculated for each variable. All interval variables were tested for normality of distribution using the Kolmogorov–Smirnov test. The data were presented as means and standard deviation in the paper. Values sampled from normal distributions were appropriately compared using a Student's *t*-test. Values without normal distributions were compared by using the nonparametric Mann–Whitney *U* test. Qualitative variables were expressed in percentage and were compared using the chi-square test and Fisher's exact test. *P* values less than 0.05 were considered significant.

## 3. Results

**Figure 1:**
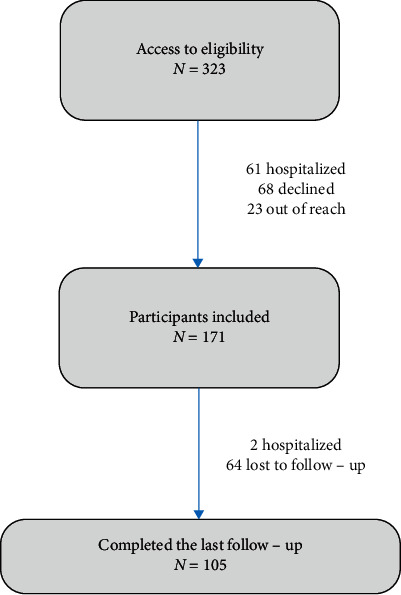
Study diagram.

A total of 105 patients were included in the study (Figure 1), most of whom were female (66.7%). Thirty-five patients (33.3%) had a history of contact with suspected COVID-19 patients, and close contact with family members was the most common reason for infection in our study. Other patients were not sure about having had contact with a known or suspected COVID-19 patient. The mean age of the patients was 39.39 years (SD: ±15.82). Among them, 42.2% had BMI ≥25 kg/m^2^ (*n* = 45) but less than 30 kg/m^2^. Past medical history of the patients showed that 41 patients (41.9%) had at least one comorbidity. The clinical characteristics and demographic data are presented in Table 1.

**Figure 2:**
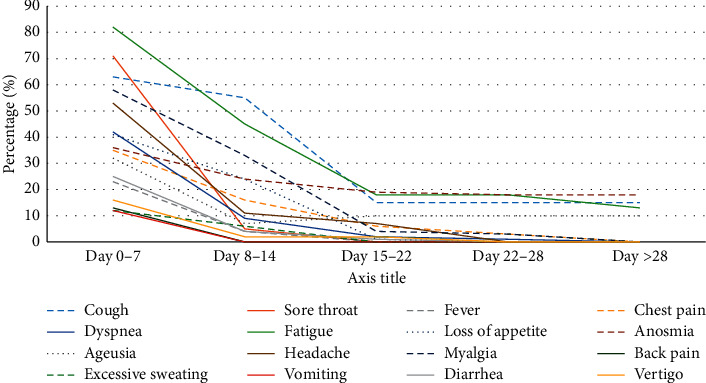
Symptom frequency during the study period.

The most common symptoms reported by patients at the time of arrival in the emergency department were cough (60%), sore throat (67%), fatigue (78.1%), headache (50.5%), and myalgia (55.2%). Only 16.2% of the patients had a temperature ≥38°C at baseline. The fever had a declining pattern in the patients on day 14, with all the patients having *T* < 37.5°C. The median time of fever resolution for most of the patients was seven days. Full details of baseline clinical symptoms are displayed in Table 1. The improvement in clinical symptoms was evaluated daily according to the patients' reported outcomes. The trend of recovery is depicted in Figure 2.

Hydroxychloroquine (HCQ) was administered for 103 (98.1%) patients according to national guidelines during the period of the study. The dosing regimen was 400 mg on the first day followed by 400 mg twice daily for five days. Additionally, 96.2% (*n* = 101) of patients received naproxen. Azithromycin and doxycycline were antibiotics consumed by patients (*n* = 72 and *n* = 33, respectively) during the quarantine. None of the patients received corticosteroids during illness. The drugs consumed by patients are reported in Table 2.

The mean scores of CRP at days 1 and 14 were 16.41 ± 16.79 and 3.76 ± 2.92, respectively, where the 14-day reduction was statistically significant (*P* < 0.001). This reduction was compatible with the recovery trend of the patients. Similar results were found for lymphocyte, leukocyte counts, and hemoglobin (*P* < 0.001). Reduction in PLR and NLR was statistically significant (*P* < 0.004, 0.001) on day 14 in comparison with day 1. All laboratory tests from the first day of admission through day 14 are shown in Table 3.

Eighteen patients were symptom-free on the first 7 days of quarantine and 32 patients on the second 7 days of quarantine.

**Figure 3:**
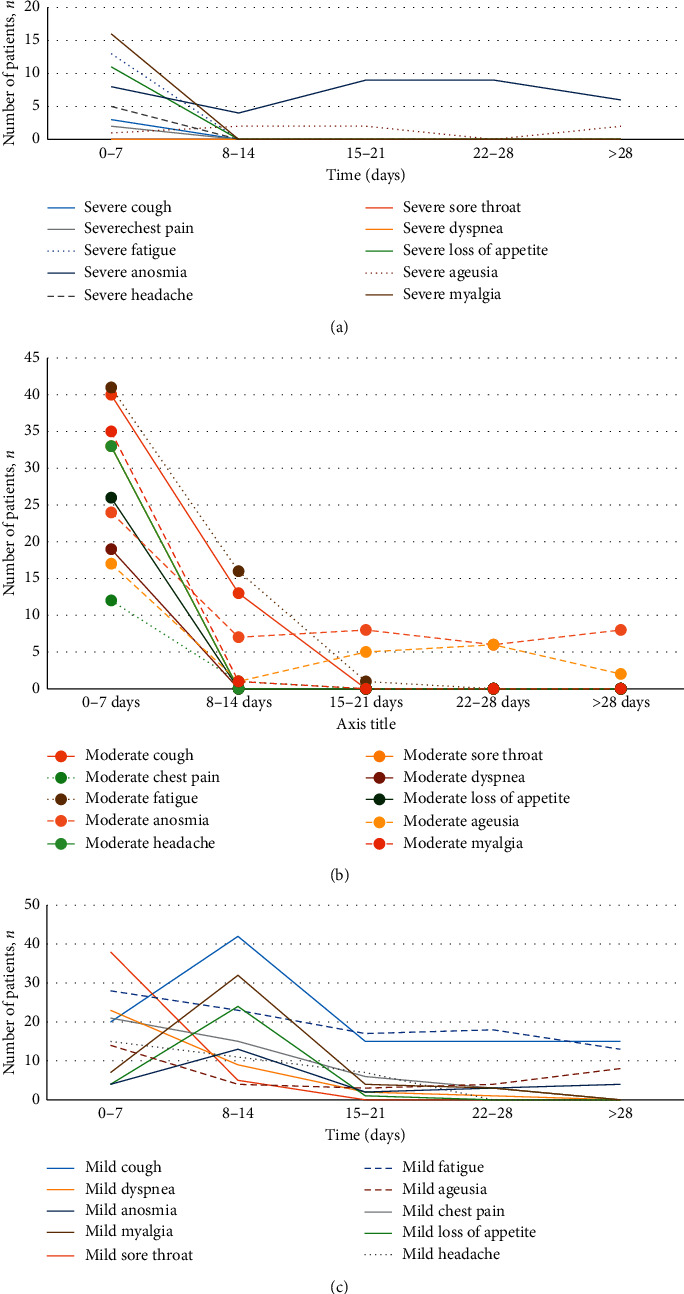
Symptom severity during the study period. (a) Severe symptoms, (b) moderate symptoms and (c) mild symptoms. Although all symptoms showed a decreasing trend during the study, most patients suffered from mild symptoms for a longer period of time.

Fifty-five patients were not symptom-free even after 14 days of quarantine. Of these, eight were required to be visited at the emergency department because of the deterioration of symptoms. The most frequent reason for revisiting included flank pain (3 patients), blood pressure fluctuations (2 patients), hemiparesis (one patient), severe weakness (one patient), and cutaneous lesions (one patient). A follow-up of the patients indicated that the severity of their symptoms decreased, but the majority did not have their prior health status after one month from the onset of symptoms. There were 40 patients (38%) with persistent symptoms at the end of the follow-up period. Most of the persistent symptoms were mild. The most common symptoms that recovered at a longer duration were anosmia (18.1%), fatigue (17.1%), and cough (14.3%) (Figure 3).

The clinical characteristics of these patients were compared with those of the recovered patients, and the results showed no significant differences between the two groups (Table 4).

## 4. Discussion

Few studies have evaluated the clinical characteristics of COVID-19 outpatients [14–16]. In this survey of 105 COVID-19 outpatients, women were more infected with mild COVID-19 than men. This is inline with the findings of a previous study where a greater proportion of nonhospitalized patients were female [15]. In addition, in another study, merely 21% of severe patients were women [16]. Our evaluated patients were younger in comparison with the admitted severe cases reported in previous studies [14, 15]. Moreover, we did not observe any patient with a BMI above 30 in the study. In fact, most of the previous studies have noted a correlation between obesity (BMI ≥ 30) and severity of the disease [17–19]. It seems obesity has an inevitable impact on infection by involving the immune system and increasing inflammatory cytokines [17]. Also, smoking history in our patients was very low, such that just three patients reported it. Similar to obesity, some previous reports have shown that smoking could be a predictive factor of disease severity [7, 8].

Nearly 42% of the patients suffered from one kind of comorbidity. In a previous study of nonhospitalized adult COVID-19 patients, the frequency was substantially higher and hypertension was the most frequent preexisting illness reported [15]. An underlying disease has been observed more in hospitalized patients than outpatients; besides, inpatients complain more about dyspnea and less about the loss of smell or taste [14].

In more than 90% of outpatients, fever and cough are the key symptoms for diagnosing COVID-19 infection [20], whereas in our study, fever just happened in one-third of the patients. Prolonged fever (*T* ≥ 38°C lasting for more than 7 days) is definitely a bad prognostic factor for a COVID-19 patient's outcome [10, 21]. In this study, the patients did not suffer from prolonged fever and were not at a risk of developing a severe kind of the disease.

Although an outpatient setting involves patients with mild symptoms, our results showed that most of the symptoms took beyond two weeks to recover after their onset. In addition, while the WHO asserts that the duration from the symptom onset to symptom recovery is 14 days [22], our results showed that nearly more than half of the patients remained symptomatic even 14–21 days after the positive SARS-CoV-2 test. This finding is consistent with the results from previous researche studies [10, 21, 23]. Based on our results, fatigue, anosmia, ageusia, and cough were the most commonly reported symptoms, which persisted for three to four weeks. Prolonged fatigue and anosmia with moderate to severe scores were the most frequent causes leading to discomfort in patients, which is inline with the findings of previous studies [10, 14].

Some unusual symptoms were reported by patients including vaginal herpetic lesions (one patient), frequent urination (nine patients), blood pressure fluctuations (three patients), and new-onset renal calculi (6 patients). Some of these symptoms, for example, urinary symptoms and herpes virus coinfection, were reported in a recent case series [24–26]. Due to lack of sufficient evidence on an effective treatment for mild COVID-19 [27, 28], most previous trials in outpatient settings have been focused on clinical characteristics and epidemiologic factors rather than therapeutic regimens [14, 15, 19–21]. However, the results of a large national cohort study of 7,295 patients with mild COVID-19 showed that the early administration of HCQ is associated with a lower rate of hospitalization and mortality as well as the absence of serious complications [29]. Similarly, HCQ was administered for almost all patients in the study according to the national guideline. It was tolerated well except in one patient who experienced palpitation, nausea, and tinnitus. The complications subsided upon drug discontinuation.

Although we did not have a comparator group, the pharmacist-based monitoring system had beneficial effects in terms of reminding the patient of the self-isolation importance in preventing the spread of the infection. It can be noted that the pharmacist consultant during home quarantine could be worthwhile to reassure the patients of their health conditions and reduce unnecessary visits to medical centers. These data were similar to those of a study that drew upon a virtual care-monitoring program [30].

There are some limitations in the study including lack of definite diagnosis of COVID-19 according to limited access to RT-PCR in some nonhospitalized patients. The second limitation was patient-reported symptoms, which were not evaluated by a physician. Another limitation concerned the dependency of the patient monitoring program on the smart phone and the Internet. Therefore, people who could not make a call were not evaluated.

## 5. Conclusion

Nonhospitalized patients with COVID-19 can experience a wide range of symptoms that may persist beyond a month. Therefore, a regular monitoring program can be vital to assess the severity and effects of these persistent symptoms on the patients' clinical outcomes and the need for medical care.

## Figures and Tables

**Table 1:** Demographic and baseline clinical characteristics.

Parameters	
Age (yr), mean (SD)	39.39 (15.82)
*Sex*	
Male, *n* (%)	35 (33.3)
Female, *n* (%)	70 (66.7)
BMI, mean (SD)	24.57 (2.7)

*Comorbidity*	
At least one comorbidity, *n* (%)	44 (41.9)
Smoking, *n* (%)	3 (2.9)
Addiction	1 (0.9)
DM, *n* (%)	9 (8.6)
HTN, *n* (%)	11 (10.5)
DLP, *n* (%)	10 (9.5)
IHD, *n* (%)	2 (1.9)
Thyroid disorder, *n* (%)	8 (7.6)
Depression, *n* (%)	2 (1.9)
Asthma, *n* (%)	6 (5.7)
Sinusitis, *n*(%)	3 (2.8)
IBD, *n* (%)	2 (1.9)
Allergy history, *n* (%)	10 (9.5)
AD, *n* (%)	1 (0.9)

*Clinical symptoms*	
Fever, *n* (%)	37 (35.2)
Sore throat, *n* (%)	71 (67.6)
Cough, *n* (%)	63 (60)
Chest pain, *n* (%)	35 (33.3)
Dyspnea, *n* (%)	42 (40)
Runny nose, *n* (%)	7 (6.7)
Fatigue, *n* (%)	82 (78.1)
Headache, *n* (%)	53 (50.5)
Myalgia, *n* (%)	58 (55.2)
Vertigo, *n* (%)	16 (15.2)
Dyspepsia, *n* (%)	5 (17)
Diarrhea, *n* (%)	25 (23.8)
Nausea, *n* (%)	12 (11.4)
Loss of appetite, *n* (%)	41 (39)
Anosmia, *n* (%)	36 (34.3)
Ageusia, *n* (%)	32 (30.5)
Excessive sweeting, *n* (%)	12 (11.4)
Skin rash, *n* (%)	5 (4.8)
Red eyes, *n* (%)	4 (3.8)

*COVID-19 data*	
COVID-19 contact history	
Travel history, *n* (%)	2 (1.9)
Family member, *n* (%)	26 (24.8)
Attending a party, *n* (%)	8 (7.6)
Unknown, *n* (%)	69 (65.7)

*COVID-19 confirmation*	
Lung CT scan involvement, *n* (%)	105 (100)
RT-PCR, *n* (%)	41 (39)

BMI: body mass index; DM: diabetes mellitus; HTN: hypertension; DLP: dyslipidemia; IHD: ischemic heart disease; IBD: irritable bowel disease; AD: Alzheimer disease; RT-PCR: reverse transcription polymerase chain reaction; CT: computerized tomography.

**Table 2:** Therapeutic agents administered during the study.

Administered drugs	*n* (%)
Statin	8 (7.6)
Naproxen	101 (96.2)
Hydroxychloroquine	103 (98.1)
Azithromycin	72 (68.6)
Doxycycline	33 (31.4)
Ceftriaxone	7 (6.7)
Vitamin D3	57 (54.3)
Zinc	6 (5.7)
Pantoprazole	48 (43.8)
Diphenhydramine	99 (94.3)
Metformin	5 (4.8)
ARBs	10 (9.5)
Anticoagulants	6 (5.7)
Beta blockers	1 (0.9)
CCBs	1 (0.9)

ARBs: angiotensin II receptor blockers; CCBs: calcium channel blockers.

**Table 3:** Comparison of laboratory findings between baseline and day 14.

Lab test	Baseline	Day 14	*P*-value
CRP, mean (SD)	16.41 (16.79)	3.76 (2.92)	<0.001
^*∗*^WBC	5000(2280)	6100 (1620)	<0.001
^*∗*^Lymphocyte	1457 (923.5)	2340 (748)	<0.001
Neutrophil	3120 (1462.5)	3392 (1216)	0.185
^*∗*^Platelet	37.5 (4.55)	38.10 (4.05)	0.005
Platelet-to-lymphocyte ratio, mean (SD)	2.220 (1.86)	1.420 (0.58)	<0.001
Neutrophil-to-lymphocyte ratio, mean (SD)	123.45 (89.07)	112.21 (59)	0.004
^*∗*^Hemoglobin	11.8 (1.10)	12 (1.30)	<0.001
Hematocrit	37.5 (4.55)	38.10 (4.05)	0.005
LDH, mean (SD)	347.48 (92.26)	341.60 (76.47)	0.402

^*∗*^Values are expressed as median (minimum-maximum). CRP: C-reactive protein; LDH: lactate dehydrogenase; SD: standard deviation.

**Table 4:** Demographics and laboratory data assessment of according to symptom recovery.

Parameters	Recovered-symptoms group (*n* = 50)	Persisted-symptoms group (*n* = 55)	*P*-value
Age (yr), mean (SD)	39.58 (15.33)	39.21 (16.4)	0.908

*Sex*			
Male, *n*	29	41	0.072
Female, *n*	21	14	
BMI, mean (SD)	24.55 (2.78)	24.59 (2.69)	24.57 (2.7)

*Comorbidity*			
Smoking, *n* (%)	0	3 (5.5)	1.0
Addiction, *n* (%)	0	1 (1.9)	1.0
DM, *n* (%)	5 (10)	4 (7.3)	0.73
HTN, *n* (%)	6 (12)	5 (9.1)	0.75
DLP, *n* (%)	8 (16)	2 (3.6)	0.05
IHD, *n* (%)	1 (2)	1 (1.8)	1.0
Thyroid disorder, *n* (%)	3 (6)	5 (9.1)	0.72
Depression, *n* (%)	2 (4)	0	0.22
Alzheimer, *n* (%)	1 (2)	0	0.48
Asthma, *n* (%)	2 (4)	4 (7.3)	0.68
Sinusitis, *n*(%)	2 (4)	1 (1.8)	0.60
IBD, *n* (%)	1 (2)	1 (1.8)	1.0
Allergy history, *n* (%)	6 (12)	4 (7.3)	0.51
Symptom onset (days), mean (SD)	5.62 (1.52)	5.58 (2.02)	0.91

*Lab tests*			
CRP, mean (SD)	18.4 (21.4)	14.61 (10.94)	0.44
WBC, mean (SD)	5792.92 (1981.9)	5736.72 (2597.7)	0.41
Lymphocyte, mean (SD)	1577.18 (896.17)	1534.12 (78.32)	0.79
Neutrophil, mean (SD)	3690.36 (1638.6)	3634.85 (1869.85)	0.44
Platelet, mean (SD)	195640 (6119.1)	187963.64 (73705.1)	0.56
Platelet-to-lymphocyte ratio, mean (SD)	202.04 (371.53)	146.82 (86.86)	0.38
Neutrophil-to-lymphocyte ratio, mean (SD)	4.58 (12.19)	2.74 (1.58)	0.87
Hemoglobin	11.98 (0.9)	12.3 (1.09)	0.86

BMI: body mass index; DM: diabetes mellitus; HTN: hypertension; DLP: dyslipidemia; IHD: ischemic heart disease; IBD: irritable bowel disease; AD: Alzheimer disease; SD: standard deviation.

## Data Availability

The data are available from the corresponding author on rational request.
